# Vital Signs: Prevalence of Doctor-Diagnosed Arthritis and Arthritis-Attributable Activity Limitation — United States, 2013–2015

**DOI:** 10.15585/mmwr.mm6609e1

**Published:** 2017-03-10

**Authors:** Kamil E. Barbour, Charles G. Helmick, Michael Boring, Teresa J. Brady

**Affiliations:** 1 Arthritis Program, Division of Population Health, National Center for Chronic Disease Prevention and Health Promotion, CDC.

## Abstract

**Background:**

In the United States, doctor-diagnosed arthritis is a common and disabling chronic condition. Arthritis can lead to severe joint pain and poor physical function, and it can negatively affect quality of life.

**Methods:**

CDC analyzed 2013–2015 data from the National Health Interview Survey, an annual, nationally representative, in-person interview survey of the health status and behaviors of the noninstitutionalized civilian U.S. adult population, to update previous prevalence estimates of arthritis and arthritis-attributable activity limitations.

**Results:**

On average, during 2013–2015, 54.4 million (22.7%) adults had doctor-diagnosed arthritis, and 23.7 million (43.5% of those with arthritis) had arthritis-attributable activity limitations (an age-adjusted increase of approximately 20% in the proportion of adults with arthritis reporting activity limitations since 2002 [p-trend <0.001]). Among adults with heart disease, diabetes, and obesity, the prevalences of doctor-diagnosed arthritis were 49.3%, 47.1%, and 30.6%, respectively; the prevalences of arthritis-attributable activity limitations among adults with these conditions and arthritis were 54.5% (heart disease), 54.0% (diabetes), and 49.0% (obesity).

**Conclusions and Comments:**

The prevalence of arthritis is high, particularly among adults with comorbid conditions, such as heart disease, diabetes, and obesity. Furthermore, the prevalence of arthritis-attributable activity limitations is high and increasing over time. Approximately half of adults with arthritis and heart disease, arthritis and diabetes, or arthritis and obesity are limited by their arthritis. Greater use of evidence-based physical activity and self-management education interventions can reduce pain and improve function and quality of life for adults with arthritis and also for adults with other chronic conditions who might be limited by their arthritis.

## Introduction

In the United States, doctor-diagnosed arthritis is a common and widespread chronic condition (*1*,*2*). Arthritis is a leading cause of disability (*3*) and is projected to affect 78.4 million adults by 2040 (*4*). The most common form of arthritis is osteoarthritis; other forms include, but are not limited to, rheumatoid arthritis, gout, lupus, and fibromyalgia. The annual direct medical costs attributable to arthritis are approximately $81 billion in the United States (*5*). About one million knee and hip joint replacements occur each year; 99% occur because of arthritis-related pain and functional limitations (*6*). Among adults with arthritis, 27% report severe joint pain (*7*); one third of adults aged ≥45 years report anxiety or depression (*8*); three in 10 find stooping, bending, or kneeling very difficult; approximately 20% cannot or find it very difficult to walk three blocks (approximately one quarter mile) or push/pull large objects (*9*). Adults with arthritis are more than twice as likely to report an injury related to a fall (*10*), and working-aged adults with arthritis have lower employment rates compared with adults without arthritis (*5*).

Arthritis is a common comorbid condition among adults with heart disease, diabetes, or obesity, and the combination of arthritis and one of these chronic conditions has been found to be associated with greater levels of physical inactivity (*11*). Moreover, arthritis may also hinder the ability of adults with prediabetes to engage in the physical activity recommended to prevent diabetes (*12*).

Many adults with arthritis are prescribed opioids (*13*), but safer options exist to help manage arthritis-associated pain. The CDC Guideline for Prescribing Opioids for Chronic Pain states that insufficient evidence for and serious risks associated with long-term use of opioid therapy to treat chronic pain exist, and recommends use of exercise therapy, cognitive behavioral therapy, certain interventional procedures, acetaminophen, and nonsteroidal anti-inflammatory drugs for the treatment of arthritis (*14*). Although medications can help, nonpharmaceutical measures help as well. For example, physical activity decreases pain and improves function, each by almost 40% (*15*), and self-management education interventions produce improvements in a person’s confidence and skills to manage their condition and can reduce pain, fatigue, and depression by 10% to 20% (*16*). However, self-management education interventions are underused by adults with arthritis; only about 11% reported ever having taken a course (*17*). Furthermore, approximately one in three adults with arthritis report no leisure-time physical activity (*18*). A health care provider’s recommendation to patients with arthritis is important, because adults with arthritis are significantly more likely to attend a self-management education program to learn to manage their condition when recommended by a provider than adults with arthritis who were not recommended (*19*,*20*). Physical activity programs can reduce yearly health care costs. For example, an analysis found that participation in EnhanceFitness,* an evidence-based physical activity intervention, reduced total health care costs by $945 per person (*21*), and produced substantial improvements (up to 18%) in function (e.g., muscle strength and balance) and self-reported health at follow-up at 8 months (*22*).

To update prevalence estimates of arthritis and arthritis-attributable activity limitations, CDC analyzed 2013–2015 data from the National Health Interview Survey (NHIS).

## Methods

NHIS is an annual, nationally representative, in-person interview survey of the health status and behaviors of the noninstitutionalized civilian U.S. adult population. In each household identified, one adult is randomly selected in each family to complete the “sample adult” questionnaire.^†^ Sampling weights were applied to account for household nonresponse and oversampling of non-Hispanic blacks (blacks), Hispanics, and non-Hispanic Asians (Asians). Poststratification adjustments were applied by the National Center for Health Statistics using 2010 census estimates for the years 2013–2015. NHIS data from 2013 (N = 34,557), 2014 (36,697), and 2015 (33,672) were combined and weighted. Annualized unadjusted and age-adjusted prevalence estimates (standardized to the projected 2000 U.S. standard population) (*23*) were calculated overall and stratified by selected demographic (sex, age group, race/ethnicity, education level, and employment status) and health (body mass index category,^§^ physical activity level,^¶^ health status, doctor-diagnosed heart disease,** and doctor-diagnosed diabetes) characteristics. Absolute percent differences for all comparisons to the referent subgroups within each characteristic were considered statistically significant if the 95% confidence intervals of the differences of the age-adjusted estimates did not include zero. Orthogonal linear contrasts were performed to examine trends over time since 2002 in the age-adjusted prevalence of doctor-diagnosed arthritis and arthritis-attributable activity limitations among adults with arthritis.

Having doctor-diagnosed arthritis was defined as answering “yes” to the question “Have you ever been told by a doctor or other health professional that you have some form of arthritis, rheumatoid arthritis, gout, lupus, or fibromyalgia?” That standard question, in use since 2002, was designed to incorporate key elements of the 1994 public health definition of arthritis developed by the National Arthritis Data Workgroup, which sought to capture conditions treated by a rheumatologist or considered arthritis or a rheumatic condition by health care providers. Those who responded “yes” to arthritis also were asked “Are you now limited in any way in any of your usual activities because of arthritis or joint symptoms?” Persons responding “yes” to both questions were categorized as having arthritis-attributable activity limitations.

## Results

During 2013–2015, an estimated 22.7% (54.4 million; age-adjusted prevalence = 21.0%) of all U.S. adults had doctor-diagnosed arthritis. Almost half (49.6%, 22.2 million) of adults aged ≥65 years had arthritis; 7.1% (8.0 million) of young adults (aged 18–44 years) and 29.3% (24.2 million) of middle-aged adults (aged 45–64 years) had arthritis (Table 1). The majority of adults with arthritis (59.0%, 32.2 million) were aged <65 years. The age-adjusted prevalence of arthritis was significantly higher among women (23.5%) than men (18.1%), and was significantly lower among Hispanics (15.4%) and Asians (11.8%) than non-Hispanic whites (whites) (22.6%), and among adults who completed college or higher (17.9%) than adults who had less than a high school education (21.2%). The age-adjusted prevalence of arthritis was highest among adults who were unable to work (43.9%) (Table 1). The age-adjusted prevalence of arthritis was lower among adults meeting physical activity recommendations (18.1%) than adults who reported insufficient activity (23.1%) or inactivity (23.6%), and higher among adults with fair/poor health (40.5%) than adults with very good/excellent health (15.4%) (Table 1).

**TABLE 1:** Unadjusted and age-adjusted* annualized weighted prevalence of doctor-diagnosed arthritis^†^ in the adult (aged ≥18 years) population, by selected characteristics — National Health Interview Survey, United States, 2013–2015

Demographic/Health characteristic	Unweighted sample size^§^	Annualized weighted sample size (millions)^§^	Weighted population distribution (%)	Prevalence of doctor-diagnosed arthritis		
				% (95% CI)		
				Unadjusted	Age-adjusted	APD^¶^
**Overall**	**104,784**	**239.5**	**(100)**	**22.7 (22.2 to 23.2)**	**21.0 (20.6 to 21.3)**	NA
**Age group (yrs)**						
18–44	44,928	112.1	(46.8)	7.1 (6.8 to 7.5)	NA	NA
45–64	35,165	82.6	(34.5)	29.3 (28.6 to 30.0)	NA	NA
≥65	24,691	44.8	(18.7)	49.6 (48.6 to 50.5)	NA	NA
**Sex**						
Men	46,851	115.4	(48.2)	19.1 (18.5 to 19.7)	18.1 (17.6 to 18.6)	Referent
Women	57,933	124.1	(51.8)	26.0 (25.5 to 26.6)	23.5 (23.1 to 24.0)	5.4 (4.9 to 6.0)
**Race/Ethnicity**						
White, non-Hispanic	64,108	157.0	(65.5)	26.3 (25.7 to 26.9)	22.6 (22.2 to 23.1)	Referent
Black, non-Hispanic	14,493	27.9	(11.6)	21.8 (20.9 to 22.8)	22.2 (21.4 to 23.0)	−0.4 (−1.3 to 0.4)
Hispanic	17,571	36.6	(15.3)	12.1 (11.4 to 12.8)	15.4 (14.6 to 16.1)	−7.3 (−8.2 to −6.4)
Asian, non-Hispanic	5,957	13.1	(5.5)	11.1 (10.1 to 12.2)	11.8 (10.9 to 12.8)	−10.8 (−11.9 to −9.8)
Multiple race, non-Hispanic	1,716	3.2	(1.3)	21.7 (19.1 to 24.5)	25.2 (22.7 to 27.9)	2.6 (−0.2 to 5.3)
American Indian/Alaska Native, non-Hispanic	741	1.3	(0.5)	24.6 (20.5 to 29.1)	24.4 (20.4 to 28.8)	1.7 (−2.4 to 5.9)
**Education level**						
<High school diploma	15,489	31.6	(13.3)	25.9 (24.9 to 26.8)	21.2 (21.2 to 22.6)	Referent
High school diploma	26,699	61.0	(25.6)	25.3 (24.5 to 26.1)	22.1 (21.5 to 22.8)	0.3 (−0.7 to 1.2)
At least some college	32,073	73.7	(30.9)	22.8 (22.1 to 23.5)	22.8 (22.2 to 23.4)	0.9 (0.0 to 1.7)
Completed college or greater	30,054	72.0	(30.2)	19.1 (18.4 to 19.8)	17.9 (17.4 to 18.5)	−3.9 (−4.9 to −3.0)
**Employment status**						
Employed/Self-employed	61,427	147.7	(61.7)	14.9 (14.5 to 15.4)	17.7 (17.2 to 18.2)	Referent
Unemployed	5,577	13.6	(5.7)	14.3 (13.1 to 15.5)	19.3 (17.6 to 21.1)	1.7 (−0.2 to 3.5)
Unable to work	8,241	16.5	(6.9)	52.0 (50.3 to 53.7)	43.9 (42.2 to 45.7)	26.2 (24.5 to 28.0)
Other**	29,491	61.6	(25.7)	35.4 (34.5 to 36.2)	21.1 (20.4 to 21.7)	3.4 (2.6 to 4.2)
**Physical activity**						
Meeting recommendations	49,063	115.8	(49.2)	17.3 (16.7 to 17.9)	18.1 (17.6 to 18.6)	Referent
Insufficient activity	20,398	46.9	(19.9)	26.0 (25.1 to 26.9)	23.1 (22.4 to 23.9)	5.1 (4.2 to 5.9)
Inactive	33,565	72.5	(30.9)	29.2 (28.5 to 30.0)	23.6 (23.1 to 24.2)	5.6 (4.8 to 6.3)
**Health status**						
Very good/Excellent	60,381	145.1	(60.6)	14.5 (14.0 to 14.9)	15.4 (15.1 to 15.8)	Referent
Good	28,719	63.3	(26.5)	28.1 (27.3 to 29.0)	23.8 (23.2 to 24.5)	8.4 (7.7 to 9.1)
Fair/Poor	15,642	31.0	(13.0)	50.0 (48.9 to 51.2)	40.5 (39.3 to 41.7)	25 (23.7 to 26.4)
**Body mass index**						
Underweight/Normal weight	36,317	84.3	(36.5)	16.4 (15.8 to 16.9)	16.4 (16.0 to 16.9)	Referent
Overweight	34,617	79.1	(34.2)	22.5 (21.8 to 23.2)	19.7 (19.1 to 20.2)	3.2 (2.6 to 3.9)
Obese	30,240	67.8	(29.3)	30.6 (29.7 to 31.4)	27.7 (27.0 to 28.4)	11.3 (10.5 to 11.5)
**Heart disease**						
No	91,280	211.6	(88.4)	19.2 (18.8 to 19.6)	19.1 (18.8 to 19.5)	Referent
Yes	13,387	27.6	(11.6)	49.3 (48.1 to 50.5)	36.4 (34.9 to 38.0)	17.3 (15.7 to 18.9)
**Diabetes**						
No	93,715	217.1	(90.7)	20.2 (19.7 to 20.7)	19.8 (19.4 to 20.2)	Referent
Yes	11,044	22.4	(9.3)	47.1 (45.8 to 48.4)	33.7 (32.0 to 35.4)	13.9 (12.1 to 15.6)

**Abbreviations:** APD = absolute percent difference; CI = confidence interval; NA = not applicable.

* Age adjusted to the 2000 U.S. projected adult population, using three age groups: 18–44, 45–64, and ≥65 years.

^†^ Doctor-diagnosed arthritis was defined as an affirmative response to the question “Have you ever been told by a doctor or other health professional that you have some form of arthritis, rheumatoid arthritis, gout, lupus, or fibromyalgia?

^§^ Some categories might not sum to overall because of missing information on some characteristics.

^¶^ APD for age-adjusted estimates.

** Students, volunteers, homemakers, retirees.

**FIGURE 1:**
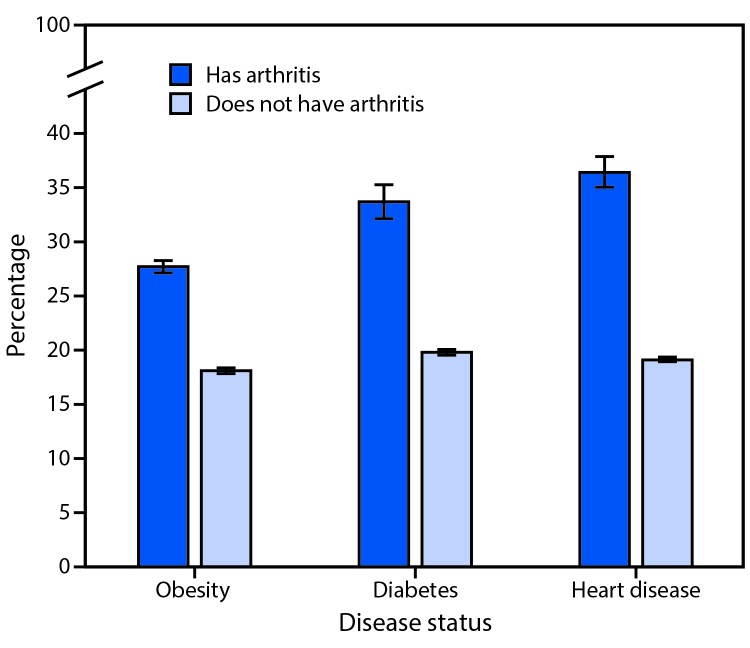
Age-adjusted percentage* of adults with doctor-diagnosed arthritis, by obesity, diabetes, and heart disease status — National Health Interview Survey, United States, 2013–2015 * With 95% confidence intervals indicated by error bars.

The unadjusted prevalences of arthritis among adults with obesity, heart disease, or diabetes were 30.6%, 49.3%, and 47.1%, respectively (Table 1) After adjustment for age, adults who had obesity compared with no obesity, had diabetes compared with no diabetes, or had heart disease compared with no heart disease were approximately 1.5, 1.7, and 1.9 times more likely to have arthritis, respectively (Figure 1).

Among adults with arthritis, an estimated 43.5% (23.7 million; age-adjusted prevalence = 41.7%) had arthritis-attributable activity limitations (Table 2). The age-adjusted prevalence of arthritis-attributable activity limitations was higher among women (43.8%) than men (38.5%); among blacks (48.6%), Hispanics (44.3%), and non-Hispanic multiracial adults (50.5%) than among whites (40.1%); among adults with less than a high school education (52.1%) than among adults with higher education (32.1%–43.6%); and among adults who were unable to work (80%) or unemployed (48.4%) than among adults who were employed (28.3%) (Table 2). The age-adjusted prevalence of arthritis-attributable activity limitations was higher among adults who were physically inactive (54.0%) than adults meeting recommendations (30.1%); among adults with fair/poor health (70.6%) than adults with very good/excellent health (23.2%); and among adults who had obesity (45.2%), heart disease (54.8%), and diabetes (52.5%) (Table 2).

**TABLE 2:** Unadjusted and age-adjusted* annualized weighted prevalence of arthritis-attributable activity limitation among adults with doctor-diagnosed arthritis,^†^ by selected characteristics — National Health Interview Survey, United States, 2013–2015

Demographic/Health characteristic	Unweighted sample size^§^	Annualized weighted sample size (millions)^§^	Weighted distribution of characteristic (%)	Prevalence of arthritis-attributable activity limitations		
				% (95% CI)		
				Unadjusted	Age-adjusted	APD^¶^
**Overall**	**26,442**	**54.3**	**(100)**	**43.5 (42.6 to 44.4)**	**41.7 (40.3 to 43.1)**	NA
**Age group (yrs)**						
18–44	3,360	8.0	(14.7)	39.4 (37.0 to 41.8)	NA	NA
45–64	10,761	24.1	(44.5)	44.5 (43.1 to 45.8)	NA	NA
≥65	12,321	22.2	(40.9)	44.0 (42.8 to 45.2)	NA	NA
**Sex**						
Men	9,740	22.0	(40.5)	40.7 (39.2 to 42.1)	38.5 (36.4 to 40.7)	Referent
Women	16,702	32.3	(59.5)	45.4 (44.5 to 46.4)	43.8 (42.2 to 45.5)	5.3 (2.7 to 7.8)
**Race/Ethnicity**						
White, non-Hispanic	18,563	41.3	(76.0)	42.1 (41.0 to 43.2)	40.1 (38.4 to 41.9)	Referent
Black, non-Hispanic	3,888	6.1	(11.2)	49.9 (47.8 to 52.0)	48.6 (45.2 to 52.0)	8.5 (4.7 to 12.2)
Hispanic	2,577	4.4	(8.1)	47.7 (45.1 to 50.3)	44.3 (41.0 to 47.6)	4.1 (0.5 to 7.8)
Asian, non-Hispanic	716	1.5	(2.7)	38.9 (34.3 to 43.7)	37.6 (27.5 to 49.0)	−2.5 (−13.5 to 8.5)
Multiple race, non-Hispanic	468	0.7	(1.3)	51.3 (44.5 to 58.0)	50.5 (41.7 to 59.2)	10.3 (1.1 to 19.5)
American Indian/Alaska Native, non-Hispanic	201	0.3	(0.6)	54.0 (44.0 to 63.6)	51.6 (37.9 to 65.0)	11.4 (−2.6 to 25.5)
**Education level**						
<High school diploma	4,634	8.2	(15.1)	55.1 (52.9 to 57.2)	52.1 (47.9 to 56.3)	Referent
High school diploma	7,421	15.4	(28.5)	44.0 (42.4 to 45.7)	43.1 (40.5 to 45.7)	−9 (−13.9 to −4.2)
At least some college	8,154	16.8	(31.1)	44.9 (43.5 to 46.4)	43.6 (41.5 to 45.7)	−8.5 (−13.2 to −3.8)
Completed college or greater	6,131	13.7	(25.4)	34.2 (32.5 to 36.0)	32.1 (29.4 to 34.8)	−20 (−25.0 to −15.1)
**Employment status**						
Employed/Self-employed	9,600	22.0	(40.5)	28.3 (27.1 to 29.6)	28.3 (26.8 to 29.9)	Referent
Unemployed	915	1.9	(3.6)	47.5 (43.2 to 51.7)	48.4 (43.8 to 53.1)	20.1 (15.2 to 25.0)
Unable to work	4,511	8.6	(15.8)	81.3 (79.6 to 82.8)	80.0 (76.9 to 82.8)	51.7 (48.5 to 54.9)
Other**	11,411	21.8	(40.1)	43.7 (42.5 to 44.9)	44.9 (40.8 to 49.1)	16.6 (12.4 to 20.8)
**Physical activity**						
Meeting recommendations	9,380	20.0	(37.5)	30.1 (28.9 to 31.4)	30.1 (28.4 to 31.9)	Referent
Insufficient activity	5,804	12.2	(22.8)	43.8 (42.0 to 45.7)	43.0 (40.2 to 45.8)	12.9 (9.6 to 16.1)
Inactive	10,800	21.2	(39.7)	55.8 (54.4 to 57.1)	54.0 (51.4 to 56.6)	23.9 (20.8 to 27.0)
**Health status**						
Very good/Excellent	9,632	21.0	(38.6)	23.4 (22.3 to 24.6)	23.2 (21.6 to 24.9)	Referent
Good	8,729	17.8	(32.8)	42.4 (41.0 to 43.8)	41.3 (38.8 to 43.7)	18.1 (15.0 to 21.2)
Fair/Poor	8,072	15.5	(28.6)	72.0 (70.6 to 73.4)	70.6 (68.0 to 73.2)	47.4 (44.5 to 50.4)
**Body mass index**						
Underweight/Normal weight	6,770	13.8	(26.3)	39.7 (38.2 to 41.3)	39.3 (36.6 to 42.0)	Referent
Overweight	8,514	17.8	(34.0)	39.7 (38.2 to 41.2)	38.7 (36.5 to 41.0)	−0.6 (−3.8 to 2.7)
Obese	10,172	20.7	(39.6)	49.0 (47.7 to 50.4)	45.2 (43.3 to 47.1)	5.9 (2.8 to 9.0)
**Heart disease**						
No	19,455	40.6	(74.9)	39.9 (38.8 to 40.8)	38.7 (37.2 to 40.3)	Referent
Yes	6,931	13.6	(25.1)	54.5 (53.0 to 56.1)	54.8 (50.9 to 58.6)	16 (11.8 to 20.8)
**Diabetes**						
No	21,048	43.8	(80.6)	41.0 (40.0 to 41.9)	39.9 (38.5 to 41.4)	Referent
Yes	5,386	10.5	(19.4)	54.0 (52.1 to 55.8)	52.5 (47.7 to 57.2)	12.5 (7.6 to 17.4)

**Abbreviation:** APD = absolute percent difference, CI = confidence interval; NA = not applicable.

* Age adjusted to the 2000 U.S. projected adult population, using three age groups: 18–44, 45–64, and ≥65 years.

^†^ Doctor-diagnosed arthritis was defined as an affirmative response to the question “Have you ever been told by a doctor or other health professional that you have some form of arthritis, rheumatoid arthritis, gout, lupus, or fibromyalgia? Those who answered “yes” were asked “Are you now limited in any way in any of your usual activities because of arthritis or joint symptoms?” Persons responding “yes” to both questions were defined as having arthritis-attributable activity limitations.

^§^ Some categories might not sum to overall because of missing information on some characteristics.

^¶^ APD for age-adjusted estimates.

** Students, volunteers, homemakers, retirees.

**Figure 2:**
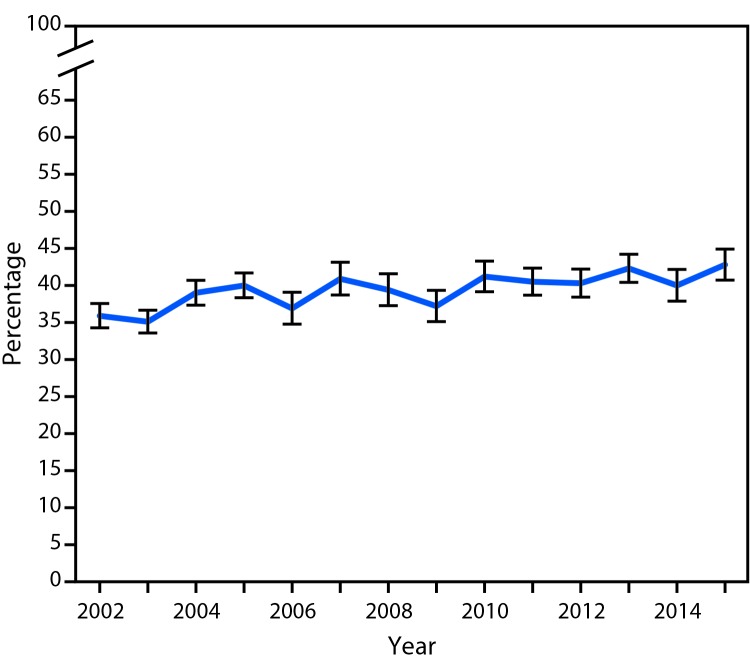
Age-adjusted percentage* of persons with arthritis-attributable activity limitations among adults with doctor-diagnosed arthritis — National Health Interview Survey, United States, 2002–2015 * With 95% confidence intervals indicated by error bars.

The age-adjusted prevalence of arthritis-attributable activity limitations among adults with arthritis was significantly higher in 2015 (42.8%; 95% CI = 40.5–45.1) compared with 2002 (35.9%; 95% CI = 34.1–37.6), an increase of 19.2% (p-trend <0.001) (Figure 2). The age-adjusted prevalence of doctor-diagnosed arthritis did not change significantly over time (p-trend = 0.71).

## Conclusions and Comments

During 2013–2015, an average of more than one in five (54.4 million) adults in the United States had doctor-diagnosed arthritis, with 43.5% (23.7 million) of adults with arthritis reporting arthritis-attributable activity limitations. The prevalence of arthritis-attributable activity limitations among adults with arthritis increased by almost 20% over time (2002–2015) independent of the aging of the U.S. population, resulting in greater pain, disability, costs, and decreased quality of life.

As found in analyses of earlier NHIS surveys (*1*), women and adults who were unable to work, with fair/poor health, or with obesity, heart disease, or diabetes, not only had a higher prevalence of arthritis, but also had a higher prevalence of arthritis-attributable activity limitations. The prevalence of arthritis among adults who were unemployed was similar to the prevalence among employed adults, but unemployed adults had a much higher prevalence of arthritis-attributable activity limitations, suggesting that arthritis-attributable activity limitations might play a role in their unemployment (*5*). Similar to past analyses, blacks and whites had a similar prevalence of arthritis, but the prevalence of arthritis-attributable activity limitations was higher among blacks. Hispanics had a much lower prevalence of arthritis, but a proportionately higher prevalence of arthritis-attributable activity limitations.

Our findings suggest that the burden of arthritis is increasing and requires more widespread use of existing, underused evidence-based interventions. Physical activity is a proven strategy for managing arthritis, with known benefits for the management of many other chronic conditions (*15*). Arthritis is common among adults with heart disease, diabetes, or obesity, and the combination of arthritis and one of these chronic conditions has been associated with higher levels of physical inactivity (*11*), suggesting that arthritis-specific barriers to physical activity (concerns about worsening pain, damaging joints, and safely exercising) might be important concerns for adults with those conditions. Improving the health of adults with arthritis, including those with these combined chronic conditions, needs to include wider dissemination and implementation of evidence-based interventions. These interventions meet the unique needs of adults with arthritis and have been found to reduce pain and improve function, mood, and confidence to manage health and quality of life (*15*,*16*).

A U.S. Department of Health and Human Services initiative addresses the effects of multiple chronic conditions,^††^ which now affect one in four adults and are becoming increasingly common as the population ages. A previous study on multiple chronic conditions among adults aged ≥25 years found that arthritis is frequently present among the most common combinations of two and three conditions (*24*). CDC is promoting greater coordination among chronic disease programs within state health departments to address these chronic disease comorbidity concerns.^§§^ Active promotion of evidence-based self-management education and physical activity interventions is appropriate for various chronic conditions. The self-management education^¶¶^ and physical activity interventions*** that CDC recommends for adults with arthritis are examples of proven, low-cost, community interventions that can benefit adults with arthritis, physical limitations, and other chronic conditions.

The findings in this report are subject to at least six limitations. First, doctor-diagnosed arthritis was self-reported and not confirmed by a health care professional; however, this case definition was validated for public health surveillance (*25*). Second, because NHIS is a cross-sectional survey, a causal relationship between risk factors (i.e., obesity or physical activity) and arthritis and arthritis-attributable activity limitations could not be established, although strong evidence exists that indicates obesity is associated with an increased risk for incident knee osteoarthritis (a common form of arthritis) (*26*). Third, social desirability bias might play a role in some self-reported characteristics, with underreporting of weight and overreporting of height and leisure-time physical activity. Fourth, from 2013 to 2015, the NHIS response rates were 61.2%, 58.9%, 55.2%, respectively, indicating potential nonresponse bias, although survey weights were applied to address this bias and improve external validity (*27*). Fifth, if multivariate analyses were to be performed, certain observed group differences, such as those related to race/ethnicity and arthritis-attributable activity limitations, might have been explained by differences in prevalence of comorbid conditions or other factors (e.g., health care access). Finally, NHIS does not survey persons in long-term care institutions (e.g., nursing homes for the elderly and hospitals for the chronically ill or physically or intellectually disabled); therefore, this analysis likely underestimates the prevalence of arthritis and arthritis-attributable activity limitations.

Arthritis is a large and growing clinical and public health problem. In 2017, CDC is funding arthritis programs in 12 states to disseminate arthritis-appropriate evidence-based physical activity and self-management education interventions in their communities.^†††^ Given the high prevalence of arthritis and the increase in arthritis-attributable activity limitations in the United States, health care providers and public health practitioners can address arthritis and other chronic conditions by prioritizing proven, nonpharmaceutical interventions, such as self-management education and appropriate physical activity, as effective ways to improve health outcomes, especially for groups with the highest prevalence of arthritis and arthritis-attributable activity limitations.

**Key Points**

• Arthritis is common, expensive, and a leading cause of disability. An estimated 54.4 million adults (22.7%) had doctor-diagnosed arthritis.

• Approximately 24 million adults with arthritis had activity limitations attributable to arthritis. Among adults with arthritis, the percentage limited by arthritis has increased by almost 20% over time.

• Approximately half of all adults with heart disease or diabetes had arthritis. Nearly one third of adults who with obesity also had arthritis. Arthritis makes managing these conditions harder.

• Adults with arthritis are often prescribed opioids in the United States; however, better ways to help manage arthritis often exist. For example, physical activity can reduce pain and improve physical function by approximately 40%. However, one in three adults with arthritis report no leisure time physical activity.

• Using confidence and skills learned in self-management education workshops can help reduce pain, fatigue, and depression by 10% to 20%. However, only 11% had taken a self-management education workshop.

• Health care providers can play an important role in the management of arthritis. For example, adults with arthritis are more likely to attend a self-management education program when it is recommended by a health care provider.

• Additional information is available at https://www.cdc.gov/vitalsigns.

## Footnotes

* https://www.ncoa.org/resources/program-summary-enhancefitness/.

† Survey description documents are available at https://www.cdc.gov/nchs/nhis/quest_data_related_1997_forward.htm.

§ Body mass index = weight (kg)/(height [m])^2^; categorized as underweight/normal weight (<25.0), overweight (25.0 to <30.0), or obese (≥30.0).

¶ Determined from responses to six questions regarding frequency and duration of participation in leisure-time activities of moderate or vigorous intensity and categorized according to the U.S. Department of Health and Human Services 2008 Physical Activity Guidelines for Americans. Total minutes (moderate to vigorous) of physical activity per week were categorized as follows: meeting recommendations (≥150 min per week); insufficient activity (1–149 min); or inactive (0 min).

** Adults were considered to have doctor-diagnosed heart disease if they answered yes to any of the following four questions: “Have you ever been told by a doctor or other health professional that you had coronary heart disease? Angina, also called angina pectoris? A heart attack (also called myocardial infarction)? Any kind of heart condition or heart disease (other than the ones I just asked about)?”

†† https://www.hhs.gov/ash/about-ash/multiple-chronic-conditions/index.html.

§§ https://www.cdc.gov/chronicdisease/about/state-public-health-actions.htm.

¶¶ https://www.cdc.gov/arthritis/interventions/self_manage.htm.

*** https://www.cdc.gov/arthritis/pa_overview.htm.

††† https://www.cdc.gov/arthritis/state_programs/programs.
